# Estimating prevalence of avian haemosporidians in natural populations: a comparative study on screening protocols

**DOI:** 10.1186/s13071-017-2066-z

**Published:** 2017-03-06

**Authors:** Farah Ishtiaq, Megha Rao, Xi Huang, Staffan Bensch

**Affiliations:** 1 Centre for Ecological Sciences, Indian Institute of Science, Bangalore, 560012 India; 2 Molecular Ecology Evolution Lab, Department of Biology, Lund University, S-22362 Lund, Sweden

**Keywords:** Avian haemosporidians, Detection probability, *Plasmodium*, *Haemoproteus*, Intensity, *Leucocytozoon*, qPCR, Restriction enzyme-based assay, Nested PCR

## Abstract

**Background:**

Birds harbour an astonishing diversity of haemosporidian parasites. Renewed interest in avian haemosporidians as a model system has placed a greater emphasis on the development of screening protocols to estimate parasite prevalence and diversity. Prevalence estimates are often based on the molecular or blood-smear microscopy techniques. However, variation in diagnostic sensitivity among screening methodologies represents a potential source of bias that may lead to erroneous inference in comparisons of prevalence across studies. Here, we analyzed a suite of blood samples for the presence of parasites using four diagnostic tools and compared method-specific estimates of detection probability to assess the relative performance of screening strategies.

**Methods:**

We screened a total of 394 bird blood samples collected in India (*n* = 203) and Sweden (*n* = 191) for the combined presence of *Plasmodium*, *Haemoproteus* and *Leucocytozoon* with three PCR assays: (i) qPCR; (ii) restriction enzyme-based assay; and (iii) nested protocol. In addition, we examined blood smears for estimates of parasite intensity which was further screened using qPCR method to evaluate if parasite intensity shows a relationship with qPCR (Ct values). Furthermore, we used single infected samples from parasite intensities: low, medium, high, very high to establish the reproducibility in qPCR.

**Results:**

For the combined data sets from India and Sweden, detection probability for submicroscopic and low intensity infections was highest for the qPCR method, followed by the nested protocol and the restriction enzyme-based assay. For high parasite intensities, the qPCR had high PCR reproducibility, with three out of three PCR replicates being positive and with consistent Ct values across all tenfold dilution series. For parasite intensities at very low and submicroscopic samples, the qPCR was reproducible in one out of the three replicates. The intensity of parasitemia estimated from smears showed inverse relationship with Ct values in both the Indian and Swedish data sets.

**Conclusions:**

Our study highlights the importance of accounting for methodological issues to better estimate infection in parasitological studies and illustrates how a wider deployment of diagnostic tools combined with statistical approaches is needed for each study, in order to provide adequate insight into the most appropriate approach to avoid erroneous inferences.

**Electronic supplementary material:**

The online version of this article (doi:10.1186/s13071-017-2066-z) contains supplementary material, which is available to authorized users.

## Background

Parasites are important drivers of ecological and evolutionary processes in their hosts. Many studies rely on detection and quantification of parasitemia to test theories concerning sexual selection [1], reproductive success, immune response [2, 3] and host-parasite co-evolution [4]. The avian malaria parasite (*Plasmodium* spp.) and other haemosporidians (*Haemoproteus* and *Leucocytozoon* spp., Phylum Apicomplexa, Order Haemosporida) are a diverse group of vector-transmitted blood parasites which are globally distributed and found in many avian species [5, 6]. Avian malaria is caused by *Plasmodium* parasites which exhibit both asexual replication (merogony) and gametocytes in erythrocytes. *Haemoproteus* spp. cause a malaria-like disease properly referred to as haemoproteosis, and have asexual replication in fixed tissue and only gametocytes in erythrocytes. *Leucocytozoon* spp. also have asexual replication in fixed tissue and only gametocytes in the blood which can be located both in red and white blood cells. Parasite prevalence, which is the proportion of individuals parasitized in a population of hosts, is a common measure used in describing infections in epidemiological studies. The validity of many comparative studies partly hinges upon the accuracy with which prevalence is estimated; underestimation of prevalence can lead to biased ecological and epidemiological inferences. Since both host and parasite interactions occur in complex and multidimensional environment, parasite prevalence tends to vary at spatial and temporal scales either due to differences in the environmental exposure to parasites [7], or host susceptibility or resistance to infection in natural populations [8]. In addition, variation in diagnostic sensitivity among screening methodologies represents a potential source of bias that may lead to erroneous inference when comparing prevalence across studies. Therefore, accurate estimates of prevalence is a pre-requisite when exploring underlying patterns that can contribute to better understanding of the evolution of parasitism and for drawing realistic inferences in epidemiological and conservation studies.

Renewed interest in avian haemosporidians as a model system has placed a greater emphasis on the development of screening protocols to estimate parasite prevalence and diversity. Avian haemosporidian prevalence data are known to vary seasonally, between species and with the age and traits of the hosts. Therefore, accurate estimates of prevalence require sufficient sample sizes [9], application of locally optimized screening protocols [10–12] and need to account for imperfect detection to explain natural variation in prevalence estimates [13, 14]. Prevalence estimates are often based on the molecular or blood-smear microscopy techniques. Both techniques encounter qualitative and quantitative limitations. Polymerase chain reaction (PCR) that detects the presence of nucleic acid by amplifying small traces of DNA has significantly changed our perspectives on avian haemosporidians epidemiology [15, 16]. To date, a number of PCR assays that detect avian haemosporidian infections have been used to characterise parasite prevalence and genetic diversity across divergent host species. Even though the PCR as a technique is highly sensitive, there are a number of potential problems that have often been overlooked when reporting and comparing prevalence data. In this paper we address some of these caveats and the need for considering methodological aspects when comparing parasitological data within and between studies.

Traditionally, haemosporidian parasites of birds have been described based on the morphology of their blood stages and intensity of infection in the host. Their identification requires microscopic inspection of blood smears, from which different cell types of the parasite can be identified within host cells [8]. Blood-smear microscopy is a cost-effective way of identifying and quantifying parasites. However, microscopy reaches its limit of detection when parasitemia falls below 40 infected red blood cells per microliter of blood which equates to one parasite per 10,000 erythrocytes [17]. Most avian haemosporidian surveys involve capture of wild birds that are generally at the chronic (relatively benign) stage of infection with low levels of parasitemia [18, 19]. Birds with acute phases of infections are thought to be under-sampled, an artefact of bird sampling techniques (e.g. mist-nets) which rely on the active movement of birds [8]. Furthermore, estimates of diagnosis heavily rely on quality of the slide preparation, the number of microscope fields analyzed and observer expertise [20].

In contrast, PCR assay can be performed on hundreds of samples archived for years under varying storage conditions, and could encounter limits of detection if parasitemia falls below 0.5 infected red blood cells per microliter of blood [21], and are less constrained by technician expertise. However, PCR-based diagnostic strategies do not differentiate among the various developmental stages within infected erythrocytes; the lower limit of detection is of the order of 10^−4^–10^−5^ parasites per erythrocyte [22]. PCR-based methods as diagnostic tools for identifying sub clinical infections (chronic) are also known to underestimate the true prevalence of infection in wild bird populations [23]. In recent years, a number of PCR assays have been described that detect avian haemosporidians infections across divergent host species (see [22, 24, 25]). Using molecular techniques, over 2,000 unique genetic haemosporidians lineages (based on cytochrome *b* sequences) were described in more than 1,100 avian species distributed all over the world and are compiled into the MalAvi database [26] which facilitates quick comparison of parasite lineages for determining the host range and geographical distribution of the parasites. Based on the MalAvi database, six PCR assays, with products ranging between 479 and 533 bp, have been primarily used for defining and comparing unique lineages. The most frequently used screening assay entails a nested PCR protocol targeting the cytochrome *b* (cyt *b*) gene that has been used in 61.7% of all publications [27]. These nested PCR protocols have selective primers that amplify either 479 bp (HAEMF and HAEMR2) for *Plasmodium* and *Haemoproteus* or 480 bp (HAEMFL and HAEMR2L) for *Leucocytozoon* parasites [28]. While nested PCR are sensitive and can increase the yield and specificity of amplification of the target DNA, they have limitations too: first, as with all PCR based methods these protocols underestimate mixed infections in birds [29]. Secondly, if the DNA is degraded or copy number of parasite DNA is low, it could lead to false negatives due to a weak template may preclude the amplification of the larger DNA piece [10, 11, 30]. In recent years, quantitative PCR (qPCR) has been used which allows simultaneous detection and quantification of parasite DNA in various sources such as bird blood, tissues or vector. Compared to more traditional approaches, such as microscopy or conventional PCR, qPCR has increased accuracy and sensitivity of target DNA detection [31, 32]. Despite the advantages, the use of this quantitative method in avian haemosporidians studies has been primarily used to determine level of parasitemia [33] or for detecting specific parasite lineages [34] and very rarely as a large scale screening tool [35]. Recently, a real-time PCR assay has been described with the distinct advantage of detecting all three genera in a single reaction [33]. This approach greatly decreases screening time and provides a cost-effective protocol for identifying the infected samples [33] for which additional analyses can be applied to identify the genera and or lineage of the parasites and possible mixed infections. Given the utility of avian haemosporidians as model system to understand the host-parasite co-evolution, there is need to emphasize the importance of combining sensitive diagnostic methods with statistical approaches that account for imperfect detection to make estimates comparable across studies. Using 394 bird blood samples from 93 host species representing 40 genera, 28 families, and 10 orders, we evaluated three detection methods; restriction enzyme-based assay, nested protocol, qPCR in two independent labs in India and Sweden. The objectives of this study are: i) to evaluate the accuracy and sensitivity of different detection methods (restriction enzyme-based assay, PCR and qPCR) for three genera of avian haemosporidians; ii) to determine the qPCR efficiency across four parasite intensities based on blood smear data; and iii) how infection intensity based on blood smear data relates with qPCR (Ct values).

## Methods

### Avian blood sampling

Birds were caught using mist nets and blood from the sub-brachial wing vein was collected in SET-buffer (20–40 μl in 500 μl buffer 0.15 M NaCl, 0.05 M Tris, 0.001 M EDTA, pH 8.0) or FTA cards (Whatman®, GE Healthcare, Buckinghamshire, England) for molecular analyses. Thin smears were prepared on glass slides and then air-dried, fixed in 100% methanol and stained with Giemsa. In India, birds were sampled between April and May in Uttarakhand and between December and February in Karnataka states in 2014–2015. For the present study, we selected 203 samples including 187 that were determined to be parasite positive by blood smears, 12 that were blood smear negative but positive by the PCR based methods and 4 samples that were negative by all methods. In Sweden, the birds were captured at Lake Krankesjön (55°41′N, 13°26′E) between July and September in 2014. For the present study we selected 191 samples for which we had high quality blood smears irrespective of whether these showed evidence of infections.

### Microscopic examination

Three (India) or two (Sweden) blood slides for each individual captured were made and screened following Godfrey et al. [36]. Briefly, all slides were first examined at low magnification (50×) for approximately 100 fields and then at least 100 fields were studied at high magnification (100×) using oil immersion lens. The microscopy of each sample took 20–25 min. The intensity of infection was recorded as per the following criterion: low (+); 1 to 10 parasites per 100 thin film fields, medium (++); 11 to 100 parasites per 100 thin film fields, high (+++); 1 to 10 parasites in one thin film field and very high (++++); more than 10 parasites in one thin film field.

### Molecular methods

DNA extractions were performed using Phenol Chloroform extraction method [37] or ammonium acetate protocol [38]. We screened all the Indian (*n* = 203) and Swedish (*n* = 191) samples for the combined presence of parasites of the genera *Plasmodium*, *Haemoproteus*, *Leucocytozoon* with three PCR assays: (i) 213 F/372R [39], a protocol that amplifies a 160 bp fragment of mitochondrial ribosomal RNA (rRNA) of avian haemosporidians designed to be followed by a restriction enzyme-based assay that identifies the parasite infection as either *Plasmodium*, *Haemoproteus* or *Leucocytozoon* as well as mixed infections; (ii) a nested protocol assay for a fragment of the cyt *b* gene that uses to sets of selective primers, HAEMF and HAEMR2 for *Plasmodium* and *Haemoproteus* (479 bp) and HAEMFL and HAEMR2L for *Leucocytozoon* (480 bp) parasites [28]; and (iii) 343 F/496R [22] for a qPCR that amplifies a 153 bp fragment of mitochondrial rRNA of avian haemosporidians.

### Quantitative PCR, standard curve and performance assessment

The Indian samples were diluted to 30 ng/μl with nuclease-free water (Qiagen, Hilden, Germany) using an ND-1000 Spectrophotometer (NanoDrop Technologies, Inc., Oxfordshire, UK). Quality of DNA for all samples was verified with the nm wavelength within the range for pure DNA (260 nm/280 nm ratios mean was 1.87–1.98). This standardization procedure allows to control for the amount of host DNA, which can interfere with amplification of parasite DNA [40]. Reactions contained DyNamo ColorFlash SYBR Green qPCR Kit on an Applied Biosystems ViiA™ 7 Real-Time PCR System. The total volume of the reaction was 10.2 μl, with 5 μl of DyNAmo ColorFlash SYBR Green Mix, 0.2 μl of ROX dye, 2 μl of the primer pair 343 F/496R (2.5 mM concentration), 1 μl of nuclease and protease free water and 2 μl of the DNA template (30 ng/μl concentration) per reaction mixture. The cycling conditions for the PCR were as follows: 50 °C for 2 min and 95 °C for 15 min followed by 40 cycles of 95 °C for 20 s, 60 °C for 25 s and 72 °C for 30 s which is followed by a melt curve analysis as per the default setting in the instrument. For this experiment, samples with all threshold cycles (Cts) higher than 38 were considered negative because repeatability decreased significantly after cycle 38 (see below), with most replicates differing by more than one Ct. Each real-time run included at least two no-template-control (NTC) wells (water in place of template DNA), and control DNA isolated from whole blood of avian host infected with *P. relictum* (GRW4; accession number: DQ659553), diluted to generate a single stock, and a non-infected bird DNA as template. The *P. relictum* DNA served as a positive control and an inter-run calibrator (IRC) produced a melt curve peak at 78.5 °C as reported by Bell et al. [33]. We ran the IRC in the same position on all plates. We ran all samples, including the IRC, in duplicate. Runs were validated only if the NTC and the negative control did not exhibit fluorescence curves that crossed the threshold line and the positive control gave a fluorescence curve that crossed the threshold line within 38 cycles (Ct ≤ 38).

Following each run, we examined the results to ensure that replicates had similar Ct values and melting peaks. We calculated a collective Ct value from each pair of replicates by averaging the fluorescence of both replicate wells at every cycle to generate a common amplification plot for the sample. We considered a sample positive at a given Ct cut-off value if the collective Ct value was less than or equal to the cut-off, at least one replicate crossed the threshold below the cut-off and the second replicate crossed no >2 cycles above the cut-off. We repeated any sample with dissimilar replicates. If a sample repeatedly yielded inconsistent replicates, or if a sample repeatedly generated melting curves with multiple peaks, we concluded that the assay could not reliably detect a parasite infection. Ct values were corrected with IRC Ct values for respective plate. To check quality of DNA extraction for samples in which we did not detect infection, we amplified a small fragment (268 bp) of avian cyt-*b* DNA using primers cytb-2RC/cytb-wow [41]. In all cases, avian DNA amplification was successful.

The overall qPCR estimate of parasitemia of the Swedish samples were carried out in a Mx3005P real time PCR instrument (Stratagene, CA, USA) using a SYBR-green (Platinum SYBR-green q-PCR SuperMix-UDG, Invitrogen, NY, USA) detection method. Each reaction included 50 ng of DNA (2 μl determined to have a DNA concentration of 25 ng/μl by analyses using a NanoDrop 2000; Thermo Scientific, Wilmington, USA), 12.5 μl Supermix, 0.1 μl ROX, 0.4 μM of each primer (343 F/496R) and ddH_2_O to reach a final volume of 25 μl. Thermal cycling conditions were as follows: after the initial incubation at 50 °C for 2 min and 95 °C for 2 min, we run 43 thermal cycles (95 °C for 15 s, 57 °C for 45 s and 72 °C for 30 s), immediately followed by a melt-analysis (between 47 and 95 °C). The samples were run as duplicates, on each plate together with two NCTs. We scored samples as positives if both of the duplicates showed evidence of amplification (amplification plots crossing the fluorescence cut-off set by the software) of a specific amplicon (as determined by inspecting the melting curves). The Ct-values were scored as the average of the two samples.

### Establishing limit of detection across samples with different parasite intensity

We established a Ct cut-off value using bird samples infected with five intensities of parasite infection (submicroscopic, low +, medium ++, high +++, very high ++++) based on microscopic examination. To verify that the established Ct cut-off fell within the dynamic range over which the real-time reaction is linear, we diluted *P. relictum*, *H. belopolyski* (accession number: AF254969), parasite DNA from infected host in different intensities 10-fold over 10 logs and ran three replicates of each dilution. We defined the limit of detection (LOD) for our assay as the lowest serial concentration at which all three replicates crossed the threshold before cycle 40 [42]. Samples with Cts higher than 38 were considered negative because repeatability decreased significantly after cycle 38. We defined linear dynamic range (LDR) as the range of concentrations (highest to LOD) over which data from all replicates could be fit to a standard curve plot with an *R* ^2^ ≥ 0.985; reactions which produced standard curves that were steeper than -3.8 are indicative of inefficient amplifications and errors in the qPCR estimation.

### Establishing how infection intensity relates with qPCR

To determine the qPCR efficiency across five parasite intensities (submicroscopic, low, medium, high and very high), linear regression was used to analyse the standard curves derived from tenfold dilution series of *Plasmodium relictum*, *Haemoproteus belopolyski* samples. Finally, linear regression model was computed using parasite intensity data (results were combined from samples classified as high and very high intensities data) from single infected samples (*n* = 160) were considered as the independent variable for each sample, whereas qPCR (Ct) values were considered the response variable for each sample. Fitness of the models was tested by Normal Q-Q plots of the Pearson residuals from the model analysis. All analyses were conducted in R (R version 3.0.2, R Development Core Team 2012). Statistical significance was defined as *P* < 0.05.

**Fig. 1:**
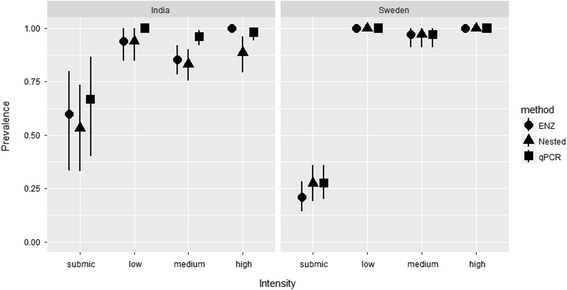
Detection methods showing discrepancy in apparent prevalence of avian haemosporidians across samples from India and Sweden with submicroscopic (submic), low, medium and high intensity based on microscopic examination. 95% CI (*black lines*) were calculated by the Sterne’s exact method (see Fig. 4 for sample size)

For comparison of prevalence data based on detection methods across different parasite intensities, using the Sterne exact method [43], we calculated unbiased haemosporidian prevalence estimates with 95% confidence intervals (95% CI) in Quantitative Parasitology, version 3.0 [44]. Prevalence estimates were considered to be significantly different if 95% CIs did not overlap (Fig. 1).

### Assessing variation in sensitivity in diagnostic methods using occupancy modelling framework

While many studies [45–48] used a single diagnostic method to obtain replicates for the estimation of detection probability, we used results from each of three diagnostic methods as replicates in an occupancy framework to estimate method-specific detection probabilities and to obtain an overall estimate of prevalence. Patterns of presence and false absence over multiple methods are used to estimate the probability of detection (ρ) of the parasite or sensitivity of the test. The apparent (or ‘naive’) prevalence is corrected for the detection probability and an unbiased estimate of the number of birds infected by the parasite (ψ) i.e. estimated prevalence, is obtained. Models containing occupancy parameters (ψ; here referred to as prevalence) and detection parameters (ρ) with covariates including intensity were used to assess variation in detection probabilities among detection methods except microscopy as intensity estimates are entirely dependent on microscopy technique. Based on four levels of parasite intensities which includes submicroscopic infections, we ran eleven models with combined datasets of India and Sweden to estimate if method-specific detection probabilities in India differs from those of Sweden given the known intensities levels. In the following models, we used a covariate ‘lab’ coded 0 (for Sweden) and 1 (for India), intensity of infection as a continuous variable and the diagnostic methods as categorical variables: model 1) ψ (lab) ρ (method * intensity) + (method * lab); model 2) ψ (lab) ρ (methods * intensity); model 3) ψ (lab) ρ (methods * intensity + lab); model 4) ψ (lab) ρ (methods + intensity + lab); model 5) ψ (lab) ρ (methods + intensity); model 6) ψ (lab) ρ (methods * lab); model 7) ψ (lab) ρ (intensity * lab); model 8) ψ (lab) ρ (methods); model 9) ψ (lab) ρ (methods + lab); model 10) ψ (lab) ρ (.); model 11) ψ (.) ρ (.). Goodness-of-fit of a general model (intensity and methods for ρ and lab for both ρ and ψ) was first examined using the parametric bootstrap goodness-of-fit procedure in program PRESENCE version 11.2 [49]. Moderate lack of fit was detected with evidence of overdispersion (ĉ >1) and models were corrected using quasi-AICc and multiplied standard errors for β values and parameters (ρ and ψ) by √ĉ [50]. We used the Akaike Information Criteria (AIC; see [50]), to select the best-fit model.

## Results

### Methodological comparison

**Fig. 2:**
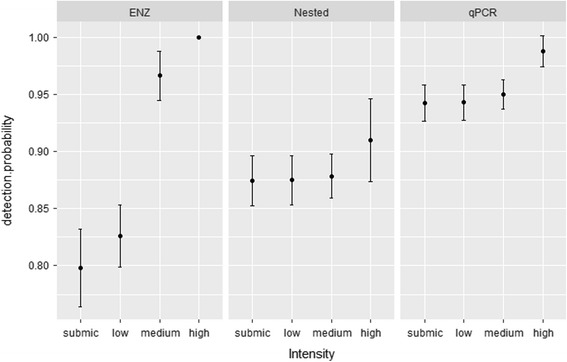
Detection methods: ENZ (restriction enzyme-based assay), Nested (nested protocol) and qPCR (quantitative PCR) showing discrepancy in detection probability (ρ) of haemosporidians across samples from submicroscopic (submic), low, medium and high intensity based on microscopic examination in combined dataset for India and Sweden for the most parsimonious model. Bars represent ± 1 SE

The apparent prevalence varied by methods for the samples from India (*n* = 203) and Sweden (191; Table 1; Fig. 1; Additional file 1: Table S1). For the combined data from India and Sweden, the best model for estimates of detection probability included the variables methods and intensity (Table 2). Detection probability for ENZ increased strongly with increasing intensity of infection (β = 20.180, standard error, SE = 7.545), although this effect was less pronounced for nested PCR (β = 0.371, SE = 0.500) and qPCR (β = 1.615, SE = 1.22) (Fig. 2). The detection probability for the nested method was slightly lower in India than in Sweden as indicated by the finding that the next best model contained the covariate lab (Table 2). Further, the overall detection probability was highest for qPCR followed by the nested and by the restriction enzyme-based methods. With increasing intensity, the detection probability does not increase significantly for the nested and qPCR methods, but increases strongly and significantly for the restriction enzyme-based method (Fig. 2).

**Table 1:** Screening of bird blood samples for haemosporidian infections. The first column shows the number of positive samples out of the total screened (203 in India^a^, 191 in Sweden^b^) for each of the methods. The columns to the right (neg) show the number of samples that were negative for the other methods

	Positive (Prevalence %)	Nested_neg_	Enz_neg_	qPCR_neg_	Smear_neg_
India					
Nested	134 (66.0)	na	7	1	7
Enz	188 (92.6)	53	na	3	10
qPCR	199 (99.0)	63	20	na	13
Smear	187 (92.1)	61	17	3	na
Total	199 (99.0)				
Sweden					
Nested	103 (53.9)	na	10	7	33
Enz	95 (49.7)	2	na	3	25
qPCR	103 (53.9)	7	11	na	33
Smear	71 (37.17)	1	1	1	na
Total	111 (58.1)				

*Abbreviations*: *Nested* nested protocol, *ENZ* restriction enzyme-based assay, *qPCR* quantitative PCR, *Smear* microscopy slides, *na* not applicable

^a^Samples with known parasite intensities based on microscopy, including 16 smear negative samples

^b^Samples selected irrespective of results by microscopy

**Table 2:** Models of occupancy (ψ), the estimate of prevalence and rho (ρ), the estimate of detection probability for haemosporidian infections assessed using three molecular detection methods on samples from India and Sweden

Model	QAIC	ΔQAIC	AICc Weights	Model Likelihood	Parameters	-2logL
Sweden + India						
{ψ (lab), ρ(methods*intensity)}	291.49	0.00	0.46	1.00	8	815.45
{ψ(lab), ρ (methods *intensity) + lab}	293.22	1.73	0.19	0.42	9	814.64
{ψ (lab), ρ (methods + intensity)}	293.58	2.09	0.16	0.35	6	833.47
{ψ (lab), ρ (methods * intensity) + (methods*lab)}	295.23	3.74	0.07	0.15	11	808.75
{ψ (lab), ρ(methods)}	296.00	4.51	0.04	0.10	5	846.57
{ψ(lab), p(methods*lab) + intensity}	296.83	5.34	0.03	0.06	9	825.33
{ψ(lab), p(methods + lab + intensity}	297.78	6.29	0.01	0.04	7	840.00
{ψ(lab), ρ(methods*lab)}	299. 59	8.10	0.00	0.00	8	839.42
{ψ (lab), ρ(methods + lab)}	301.96	10.47	0.00	0.00	6	858.28
{ψ(lab), ρ(.)}	333.92	42.43	0.00	0.00	2	976.55
{ψ(lab), ρ(intensity + lab)}	357.28	65.79	0.00	0.00	4	1033.87
{ψ(lab), ρ(intensity*lab)}	427.48	135.99	0.00	0.00	3	1247.58

### qPCR assay validation

**Fig. 3:**
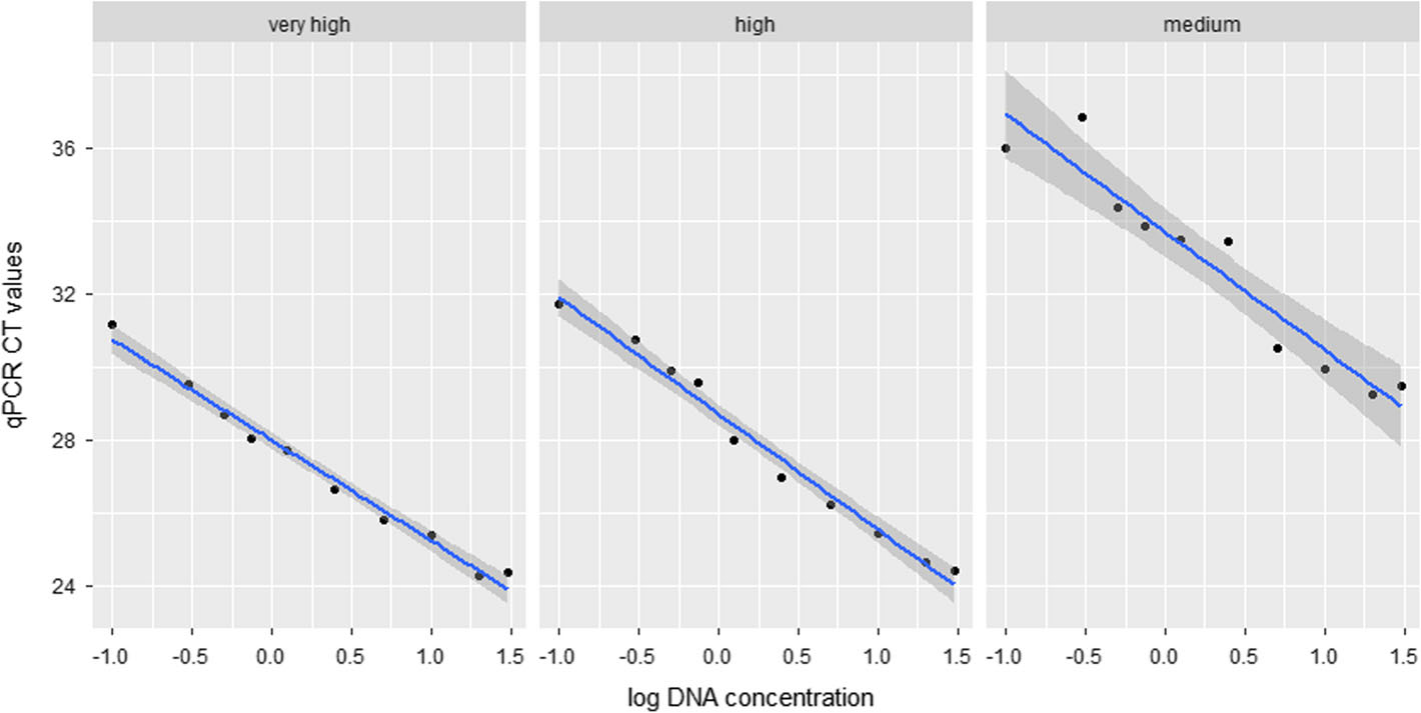
The qPCR standard curves derived from tenfold dilution series of *P. relictum* and *H. belopolyski* samples of intensity: very high (++++); high (+++); medium (++) to low (+) (see text for details). The qPCR efficiency and coefficient of determination (*R* ^*2*^) of the standard curves were calculated

**Fig. 4:**
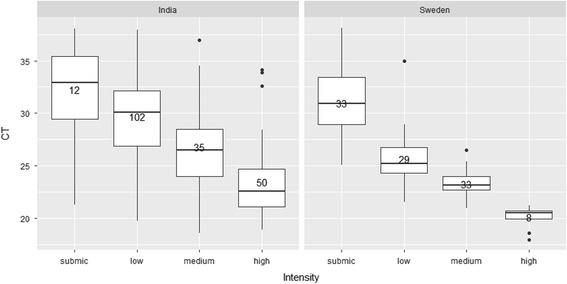
Boxplots of samples from India and Sweden showing median, upper and lower quartiles of the individual qPCR (Ct) values correlated with parasite intensity based on microscopic examination. submic (submicroscopic), low (+), medium (++), combined classes high (+++) and very high (++++)

The amplification efficiency of the qPCR for samples with high to very high parasite intensity was 98.2%, with *R* ^*2*^ values between 0.98 and 0.99, showing a linear correlation (*P* < 0.01) between the Ct values and log 10 parasite intensity (Fig. 3). Therefore, the serial dilution of the qPCR reactions for low and submicroscopic intensity samples produced standard curves that were steeper than -3.8 are indicative of inefficient amplifications and errors in the qPCR estimation. For high parasite intensities, the qPCR had high PCR reproducibility, with three out of three PCR replicates being positive and consistent Ct values across all tenfold dilution series. For parasite intensities at very low and submicroscopic samples, the qPCR was reproducible in one out of the three replicates > 1.25 ng/μl dilution. The intensity of parasitemia estimated from smears showed inverse relationship with Ct values (-3.0 + 36.07; *R* ^*2*^ = 40, *P* < 0.001) i.e. low intensity samples showed high Ct values (Fig. 4).

## Discussion

Our comparisons to evaluate the accuracy and sensitivity across three detection methods (restriction enzyme-based PCR, nested PCR and qPCR) with varying intensity for avian haemosporidians detected in samples from India and Sweden revealed qPCR as the most accurate and sensitive method especially in the case of submicroscopic (low intensity) infections, which is concordant with previous studies conducted on avian haemosporidians [35, 51] as well as human *Plasmodium* [31, 32]. However, in the Swedish data set, the sensitivity of the nested PCR did not differ from the qPCR method.

Using an occupancy modelling framework on the combined datasets from the two labs, we found the detection probability to vary by diagnostic methods and infection intensity, suggesting strong effects of the covariates. In addition, quantification of parasitemia based on microscopy or qPCR was important to understand the limit of parasite detection and enhanced the capacity of detecting submicroscopic infections. Whilst the main goal of this comparative study was to evaluate the effects of methods and intensity on parasite detection probability, there was no profound effect of ‘lab’ on the detection probability. These results highlight that the use of different screening protocols helps to reduce false negatives in the data. Taken together, our findings further highlights that it is important to consider combining screening protocols with statistical approaches in order to account for imperfect detection.

There is a need to apply a sensitive and comparable methodology across studies to minimise false comparisons and to avoid any bias in estimates of pathogen diversity [52]. Recent reviews of this rapidly emerging field have focussed primarily on the advantages and disadvantages of molecular and microscopic methods [53, 54]. However, a combination of two techniques is deemed essential for reliable estimates of prevalence across comparative studies and identification of competent hosts [20]. In terms of improving the accuracy of prevalence estimates, the combination of microscopy data and qPCR was useful in understanding the lower limit of parasite detection by relying solely on molecular versus microscopy techniques. Microscopy is a useful diagnostic tool; however as malaria prevalence decreases, microscopy may become a less useful tool if the relative proportion of submicroscopic infections increases with lower prevalence as has been shown in studies of human malaria [55]. Diagnostic screening with an additional primer assay targeting small DNA fragments needs to be considered for studies which do not combine microscopy data with molecular results. Furthermore, negative PCR results might result from low-quality or insufficient parasite DNA template which could lead to false negatives, problems associated with PCR product size, target gene copy number and PCR primer and probe binding sites [55, 56]; weak template precludes the amplification of the larger DNA piece [10, 11, 30]. The qPCR primers (343 F/496R) amplify all three parasite genera successfully (but see Bell et al. [33] reported it did not identify or match *Leucocytozoon* sequences). However, the limited differences in sequences between the primers preclude melting temperatures to be used for genus identification by this qPCR method. Nonetheless, qPCR as a diagnostic assay is bound to improve resolution of combined prevalence data. The main advantage of using 213 F/372R restriction enzyme-based assay is that it can be used to differentiate between three commonly studied parasite genera: *Plasmodium*, *Haemoproteus* and *Leucocytozoon*, and is a relatively inexpensive method [39]. There has been a strong bias towards screening only for *Plasmodium* and *Haemoproteus* but not for *Leucocytozoon*, which has remained largely understudied. This is particularly true in areas of high host diversity, where the increased cost of PCR amplifications using nested protocols can make screening for *Leucocytozoon* restrictive. Restriction enzyme-based assay offers an economical solution to screen samples for three parasite genera at no extra cost in terms of sequencing to generate prevalence data. Bentz et al. [51] proposed a similar methodology using qPCR-RFLP assay for initial screening of avian haemosporidians.

Ct values and intensity data provided a clear linear inverse relationship that implies qPCR (Ct) values are comparable for a wide range of microscopy intensity data. One potential disadvantage of qPCR methodologies for assessing parasite presence and intensity is that real time threshold cycle time (Ct) may not be directly related to parasite load. Therefore, our qPCR results, using samples of varying parasite intensity, showed a direct relation with Ct values. However, the biggest advantage of qPCR is its sensitivity to the target DNA. Whereas the traditional microscopic technique relies on semi-quantitative categorical classification of direct counts of the parasite cells (at low levels) or estimation of parasite cell counts (at high levels), the molecular technique of qPCR quantitatively “measures” the amount of target DNA present. Hence, it is important to point out that this methodology measures both DNA from living functional parasites and DNA from degraded parasites.

## Conclusions

Overall, this study highlights the significance of using various detection and quantification methods for estimating infection parameters, and the need of considering methodological aspects when comparing parasitological data within and between studies. Diagnostic sensitivities vary among methods, laboratories and study areas such that accounting for the occurrence of false negatives is critical to allow for valid comparisons of prevalence among studies.

## Additional file

Additional file 1: Table S1. List of birds and corresponding data on haemosporidian prevalence results from each screening method. (PDF 671 kb)

## Abbreviations

cytb: Cytochrome *b*
IRC: Inter-run calibrator
NTC: No-template-control
PCR: Polymerase chain reaction
qPCR: Quantitative PCR
