# Supplementary material for: Estimating prevalence of avian haemosporidians in natural populations: a comparative study on screening protocols
Source: Parasit Vectors. 2017 Mar 6;10:127. doi: 10.1186/s13071-017-2066-z (PMC5340044; doi:10.1186/s13071-017-2066-z)
Supplement: Additional file 1: Table S1. — List of birds and corresponding data on haemosporidian prevalence results from each screening method. (PDF 671 kb) [file 13071_2017_2066_MOESM1_ESM.pdf]

Estimating prevalence of avian haemosporidians in natural populations – a comparative study on screening protocols

Farah Ishtiaq[1*], Megha Rao[1], Xi Huang[2], Staffan Bensch[2]

[1]Centre for Ecological Sciences, Indian Institute of Science, Bangalore 560012, India

[2]Molecular Ecology Evolution Lab, Department of Biology, Lund University, S-22362, Lund, Sweden

Supplementary material:

INDIA

Parasite screening method

Scientific name	Family	Year	Site	mic	qPCR	enz	nested	intensity	CT value
Acrocephalus dumetorum	Acrocephaliidae	2015	Karnataka	1	1	1	1	3	24.33
Acrocephalus dumetorum	Acrocephaliidae	2015	Karnataka	1	1	1	1	3	24.14
Acrocephalus dumetorum	Acrocephaliidae	2015	Karnataka	1	1	1	1	2	28.29
Acrocephalus dumetorum	Acrocephaliidae	2015	Karnataka	1	1	1	1	2	24.92
Acrocephalus dumetorum	Acrocephaliidae	2015	Karnataka	1	1	1	1	2	27.25
Acrocephalus dumetorum	Acrocephaliidae	2015	Karnataka	1	1	1	1	3	24.22
Acrocephalus dumetorum	Acrocephaliidae	2015	Karnataka	1	1	1	1	3	22.72
Acrocephalus dumetorum	Acrocephaliidae	2015	Karnataka	1	1	1	1	2	22.21
Acrocephalus dumetorum	Acrocephaliidae	2015	Karnataka	1	1	1	1	1	27.30
Acrocephalus dumetorum	Acrocephaliidae	2015	Karnataka	1	1	1	1	2	21.10
Acrocephalus dumetorum	Acrocephaliidae	2015	Karnataka	1	1	1	1	3	27.34
Acrocephalus dumetorum	Acrocephaliidae	2015	Karnataka	1	1	1	1	3	22.61
Acrocephalus dumetorum	Acrocephaliidae	2015	Karnataka	0	1	1	1	0	21.29
Acrocephalus dumetorum	Acrocephaliidae	2014	Karnataka	1	1	1	1	1	19.77
Acrocephalus dumetorum	Acrocephaliidae	2015	Uttarakhand	1	1	1	1	1	33.16
Aegithalos concinnus	Aegithalidae	2015	Uttarakhand	1	1	1	1	1	23.51
Aegithalos concinnus	Aegithalidae	2015	Uttarakhand	1	1	1	0	3	20.32
Aegithalos concinnus	Aegithalidae	2015	Uttarakhand	1	1	1	1	2	25.25
Carpodacus erythrinus	Fringillidae	2015	Uttarakhand	0	1	0	0	0	35.05
Carpodacus erythrinus	Fringillidae	2015	Uttarakhand	1	1	0	0	1	32.18
Carpodacus erythrinus	Fringillidae	2015	Uttarakhand	1	1	0	1	1	24.73
Carpodacus erythrinus	Fringillidae	2015	Uttarakhand	1	1	1	1	3	24.70
Carpodacus erythrinus	Fringillidae	2015	Uttarakhand	1	1	1	1	1	30.56

Carpodacus erythrinus	Fringillidae	2015	Uttarakhand	1	1	1	0	1	28.63
Carpodacus erythrinus	Fringillidae	2015	Uttarakhand	1	1	1	0	1	27.42
Carpodacus erythrinus	Fringillidae	2015	Uttarakhand	1	1	1	0	1	30.21
Carpodacus erythrinus	Fringillidae	2015	Uttarakhand	1	1	1	0	1	29.42
Carpodacus erythrinus	Fringillidae	2015	Uttarakhand	1	1	1	1	1	30.73
Carpodacus erythrinus	Fringillidae	2015	Uttarakhand	1	1	1	1	1	30.11
Carpodacus erythrinus	Fringillidae	2015	Uttarakhand	0	1	0	1	0	32.64
Carpodacus rodochroa	Fringillidae	2015	Uttarakhand	0	1	1	0	0	36.80
Carpodacus rodochroa	Fringillidae	2015	Uttarakhand	1	1	0	0	1	37.95
Carpodacus rodochroa	Fringillidae	2015	Uttarakhand	1	1	1	0	1	30.49
Carpodacus rodochroa	Fringillidae	2015	Uttarakhand	1	1	1	0	1	28.04
Carpodacus rodochroa	Fringillidae	2015	Uttarakhand	1	1	1	1	3	21.06
Carpodacus rodochroa	Fringillidae	2015	Uttarakhand	1	1	1	1	1	33.30
Carpodacus rodochroa	Fringillidae	2015	Uttarakhand	1	1	1	0	1	35.69
Carpodacus rodochroa	Fringillidae	2015	Uttarakhand	1	1	1	0	1	37.48
Carpodacus rodochroa	Fringillidae	2015	Uttarakhand	1	1	1	0	1	34.39
Carpodacus rodochroa	Fringillidae	2015	Uttarakhand	1	1	1	1	1	26.01
Carpodacus rodochroa	Fringillidae	2015	Uttarakhand	1	1	1	1	3	19.99
Carpodacus rodochroa	Fringillidae	2015	Uttarakhand	1	1	1	1	3	20.78
Carpodacus rodochroa	Fringillidae	2015	Uttarakhand	1	1	1	1	3	20.56
Carpodacus rodochroa	Fringillidae	2015	Uttarakhand	1	1	1	1	3	18.96
Carpodacus rodopeplus	Fringillidae	2015	Uttarakhand	1	1	1	0	1	35.29
Carpodacus rodopeplus	Fringillidae	2015	Uttarakhand	1	1	1	0	1	29.35
Centropus sinensis	Cuculidae	2015	Karnataka	1	1	1	1	3	32.58
Centropus sinensis	Cuculidae	2014	Karnataka	1	1	1	1	1	32.85
Chrysomma sinense	Sylvidae	2015	Uttarakhand	0	1	1	1	0	32.42
Chrysomma sinense	Sylvidae	2015	Uttarakhand	1	1	1	1	1	35.37
Chrysomma sinense	Sylvidae	2015	Uttarakhand	1	1	1	1	1	31.69
Cinnyris asiaticus	Necteriniidae	2015	Uttarakhand	1	1	1	1	3	23.30

Cinnyris asiaticus	Necteriniidae	2015	Uttarakhand	1	1	1	0	1	30.60
Copsychus saularis	Muscicapidae	2015	Karnataka	1	1	1	1	1	24.98
Copsychus saularis	Muscicapidae	2015	Uttarakhand	1	1	1	1	1	31.28
Copsychus saularis	Muscicapidae	2015	Uttarakhand	1	1	1	1	1	24.61
Copsychus saularis	Muscicapidae	2015	Uttarakhand	1	1	1	1	1	26.85
Copsychus saularis	Muscicapidae	2015	Uttarakhand	1	1	1	0	1	26.82
Dendrocitta formosae	Corvidae	2015	Uttarakhand	1	1	1	1	3	21.03
Dicrurus leucophaeus	Dicruridae	2015	Uttarakhand	1	1	1	1	1	24.97
Dicrurus leucophaeus	Dicruridae	2014	Uttarakhand	1	1	1	1	3	25.56
Eumiyas thalassinus	Muscicapidae	2015	Uttarakhand	1	1	1	1	1	24.79
Ficedula parva	Muscicapidae	2015	Uttarakhand	1	1	1	0	1	33.95
Ficedula strophiata	Muscicapidae	2015	Uttarakhand	1	1	1	1	2	23.02
Ficedula superciliaris	Muscicapidae	2015	Uttarakhand	1	1	1	1	3	21.08
Ficedula tricolor	Muscicapidae	2015	Uttarakhand	1	0	1	0	1	-
Fulvetta vinipectus	Sylvidae	2015	Uttarakhand	1	0	0	0	1	-
Garrulax albogularis	Leiothrichidae	2015	Uttarakhand	1	1	1	1	1	31.71
Garrulax albogularis	Leiothrichidae	2015	Uttarakhand	1	1	1	1	1	31.98
Garrulus glandarius	Corvidae	2014	Uttarakhand	1	1	1	0	2	28.22
Glaucidium brodiei	Strigidae	2014	Uttarakhand	1	1	1	1	3	28.42
Heterophasia capistrata	Leiothrichidae	2015	Uttarakhand	0	1	1	1	0	38.02
Heterophasia capistrata	Leiothrichidae	2015	Uttarakhand	0	1	1	1	0	35.45
Heterophasia capistrata	Leiothrichidae	2015	Uttarakhand	1	1	1	0	3	23.48
Heterophasia capistrata	Leiothrichidae	2015	Uttarakhand	1	1	1	0	1	25.73
Heterophasia capistrata	Leiothrichidae	2015	Uttarakhand	1	1	1	0	2	24.97
Heterophasia capistrata	Leiothrichidae	2015	Uttarakhand	1	1	1	0	3	21.05
Heterophasia capistrata	Leiothrichidae	2015	Uttarakhand	1	1	1	1	2	22.59
Heterophasia capistrata	Leiothrichidae	2015	Uttarakhand	1	1	1	0	1	27.40
Heterophasia capistrata	Leiothrichidae	2015	Uttarakhand	1	1	1	0	2	27.40

Heterophasia capistrata	Leiothrichidae	2015	Uttarakhand	1	1	1	1	3	23.19
Leptocoma zeylonica	Necteriniidae	2015	Karnataka	1	1	1	1	1	25.86
Lonchura punctulata	Estrildidae	2015	Karnataka	1	1	1	1	2	26.77
Lonchura punctulata	Estrildidae	2015	Karnataka	1	1	1	1	3	24.67
Megalaima virens	Megalaimidae	2014	Uttarakhand	1	1	1	1	3	33.89
Megalaima virens	Megalaimidae	2014	Uttarakhand	1	1	1	1	3	34.13
Megalaima virens	Megalaimidae	2015	Uttarakhand	1	1	1	1	3	21.45
Megalaima viridis	Megalaimidae	2015	Karnataka	1	0	1	0	3	-
Megalaima viridis	Megalaimidae	2014	Karnataka	1	1	1	1	3	21.61
Megalaima viridis	Megalaimidae	2014	Karnataka	1	1	1	1	2	22.57
Muscicapa dauurica	Muscicapidae	2015	Karnataka	1	1	1	1	2	23.02
Myophonus caeruleus	Muscicapidae	2014	Uttarakhand	1	1	1	1	3	25.33
Myophonus caeruleus	Muscicapidae	2014	Uttarakhand	1	1	1	1	2	24.90
Niltava sundara	Muscicapidae	2015	Uttarakhand	1	1	1	0	1	37.81
Niltava sundara	Muscicapidae	2015	Uttarakhand	1	1	1	0	1	31.90
Niltava sundara	Muscicapidae	2015	Uttarakhand	1	1	1	0	1	31.04
Orthotomus sutorius	Cisticolidae	2015	Uttarakhand	1	1	1	1	1	27.23
Orthotomus sutoruis	Cisticolidae	2015	Karnataka	1	1	1	1	1	24.78
Orthotomus sutoruis	Cisticolidae	2014	Karnataka	1	1	0	1	1	29.07
Parus monticolus	Paridae	2015	Uttarakhand	1	1	1	0	1	37.14
Parus monticolus	Paridae	2015	Uttarakhand	1	1	1	0	1	32.93
Passer domesticus	Passeridae	2015	Uttarakhand	1	1	1	0	1	30.71
Passer rutilans	Paridae	2015	Uttarakhand	1	1	1	0	3	22.69
Passer rutilans	Paridae	2015	Uttarakhand	1	1	1	1	3	23.95
Passer rutilans	Paridae	2015	Uttarakhand	1	1	1	1	3	19.63
Phoenicurus frontalis	Muscicapidae	2015	Uttarakhand	1	1	1	0	1	30.32
Phoenicurus frontalis	Muscicapidae	2015	Uttarakhand	1	1	1	1	2	33.20
Phoenicurus frontalis	Muscicapidae	2015	Uttarakhand	0	1	1	0	0	35.38

Phylloscopus humei	Phylloscopidae	2015	Uttarakhand	1	1	1	1	3	21.10
Phylloscopus reguloides	Phylloscopidae	2015	Uttarakhand	1	1	1	1	3	23.34
Phylloscopus reguloides	Phylloscopidae	2015	Uttarakhand	1	1	1	1	3	22.58
Phylloscopus reguloides	Phylloscopidae	2015	Uttarakhand	1	1	1	1	1	27.37
Phylloscopus trochiloides	Phylloscopidae	2014	Uttarakhand	1	1	1	1	3	22.06
Phylloscopus trochiloides	Phylloscopidae	2014	Uttarakhand	1	1	0	1	1	33.05
Phylloscopus trochiloides	Phylloscopidae	2014	Uttarakhand	1	1	1	1	1	31.04
Phylloscopus trochiloides	Phylloscopidae	2015	Uttarakhand	0	1	1	0	0	38.88
Phylloscopus trochiloides	Phylloscopidae	2015	Uttarakhand	0	1	0	1	0	38.60
Phylloscopus trochiloides	Phylloscopidae	2015	Uttarakhand	1	1	1	1	3	19.89
Phylloscopus xanthoschistos	Phylloscopidae	2014	Uttarakhand	1	1	1	1	3	27.08
Phylloscopus xanthoschistos	Phylloscopidae	2015	Uttarakhand	1	1	1	1	1	29.13
Pomatorhinus erythrogenys	Timaliidae	2015	Uttarakhand	0	1	1	1	0	27.90
Pomatorhinus ruficollis	Timaliidae	2015	Uttarakhand	1	1	1	1	2	23.20
Prinia socialis	Cisticolidae	2015	Karnataka	1	1	1	0	1	27.27
Prinia socialis	Cisticolidae	2015	Karnataka	1	1	0	0	1	27.03
Prinia socialis	Cisticolidae	2015	Uttarakhand	1	1	1	1	1	30.88
Prunella strophiata	Prunellidae	2015	Uttarakhand	1	1	1	0	1	29.76
Prunella strophiata	Prunellidae	2015	Uttarakhand	1	1	1	1	1	32.16
Psilopogon zeylanicus	Megalaimidae	2015	Uttarakhand	1	1	1	1	1	30.78
Pycnonotus cafer	Pycnonotidae	2015	Uttarakhand	1	1	1	1	1	39.37
Pycnonotus cafer	Pycnonotidae	2015	Uttarakhand	1	1	1	1	1	32.43
Pycnonotus cafer	Pycnonotidae	2015	Uttarakhand	1	1	1	0	2	30.80
Pycnonotus cafer	Pycnonotidae	2015	Uttarakhand	1	1	1	0	1	34.50
Pycnonotus cafer	Pycnonotidae	2015	Uttarakhand	0	1	1	0	0	33.21
Pycnonotus cafer	Pycnonotidae	2015	Uttarakhand	1	1	1	1	2	24.53
Pycnonotus cafer	Pycnonotidae	2015	Uttarakhand	1	1	1	1	1	27.16
Pycnonotus cafer	Pycnonotidae	2015	Uttarakhand	1	1	0	1	2	36.95

Pycnonotus cafer	Pycnonotidae	2015	Uttarakhand	1	1	1	0	2	30.26
Pycnonotus cafer	Pycnonotidae	2015	Uttarakhand	1	1	1	1	1	29.50
Pycnonotus cafer	Pycnonotidae	2015	Uttarakhand	0	1	1	1	0	29.47
Pycnonotus cafer	Pycnonotidae	2015	Uttarakhand	1	1	1	1	1	32.54
Pycnonotus cafer	Pycnonotidae	2015	Uttarakhand	1	1	1	1	1	31.64
Pycnonotus cafer	Pycnonotidae	2015	Uttarakhand	1	1	1	1	2	33.04
Pycnonotus cafer	Pycnonotidae	2015	Uttarakhand	1	1	1	1	1	30.03
Pycnonotus cafer	Pycnonotidae	2015	Uttarakhand	1	1	0	1	1	36.78
Pycnonotus cafer	Pycnonotidae	2015	Uttarakhand	1	1	1	1	3	20.34
Pycnonotus leucogenys	Pycnonotidae	2015	Uttarakhand	1	1	1	0	1	30.77
Pycnonotus leucogenys	Pycnonotidae	2014	Uttarakhand	1	1	1	1	2	29.74
Pycnonotus leucogenys	Pycnonotidae	2015	Uttarakhand	0	1	0	0	0	29.35
Pycnonotus leucogenys	Pycnonotidae	2015	Uttarakhand	1	1	1	1	2	33.57
Pycnonotus leucogenys	Pycnonotidae	2015	Uttarakhand	1	1	1	1	2	34.51
Pycnonotus leucogenys	Pycnonotidae	2015	Uttarakhand	1	1	1	1	1	26.12
Pycnonotus leucogenys	Pycnonotidae	2015	Uttarakhand	1	1	1	1	2	26.53
Pycnonotus leucogenys	Pycnonotidae	2015	Uttarakhand	1	1	1	1	1	26.38
Pycnonotus leucogenys	Pycnonotidae	2015	Uttarakhand	1	1	1	0	2	26.85
Pycnonotus leucogenys	Pycnonotidae	2015	Uttarakhand	1	1	1	1	1	25.63
Pycnonotus leucogenys	Pycnonotidae	2015	Uttarakhand	1	1	1	1	1	30.78
Pycnonotus leucogenys	Pycnonotidae	2015	Uttarakhand	1	1	1	1	3	27.32
Pycnonotus leucogenys	Pycnonotidae	2015	Uttarakhand	1	1	1	0	1	27.68
Pycnonotus luteolus	Pycnonotidae	2015	Karnataka	1	1	0	0	2	27.92
Pyrrhula erythrocephala	Fringillidae	2015	Uttarakhand	1	1	1	1	1	35.31
Pyrrhula erythrocephala	Fringillidae	2015	Uttarakhand	1	1	1	1	2	25.17
Pyrrhula erythrocephala	Fringillidae	2015	Uttarakhand	1	1	1	1	1	28.80
Pyrrhula erythrocephala	Fringillidae	2015	Uttarakhand	1	1	1	0	1	35.52
Pyrrhula erythrocephala	Fringillidae	2015	Uttarakhand	1	1	0	0	1	28.17

Pyrrhula erythrocephala	Fringillidae	2015	Uttarakhand	1	1	1	1	2	27.57
Saxicola caprata	Muscicapidae	2015	Uttarakhand	1	1	1	1	1	28.68
Saxicola ferreus	Muscicapidae	2015	Uttarakhand	1	1	1	0	1	26.27
Saxicola ferreus	Muscicapidae	2015	Uttarakhand	1	1	1	0	1	38.81
Saxicola ferreus	Muscicapidae	2015	Uttarakhand	1	1	1	1	1	20.23
Seicercus whistleri	Phylloscopidae	2015	Uttarakhand	0	1	1	0	0	36.99
Tarsiger chrysaeus	Muscicapidae	2015	Uttarakhand	0	0	1	0	0	-
Trochalopteron erythrocephalus	Leiothrichidae	2014	Uttarakhand	1	1	1	1	3	21.91
Trochalopteron erythrocephalus	Leiothrichidae	2015	Uttarakhand	1	1	1	1	1	30.93
Trochalopteron erythrocephalus	Leiothrichidae	2015	Uttarakhand	1	1	1	1	1	22.66
Trochalopteron erythrocephalus	Leiothrichidae	2015	Uttarakhand	1	1	1	0	1	27.41
Trochalopteron erythrocephalus	Leiothrichidae	2015	Uttarakhand	1	1	1	0	1	24.22
Trochalopteron erythrocephalus	Leiothrichidae	2014	Uttarakhand	1	1	1	1	3	21.61
Trochalopteron erythrocephalus	Leiothrichidae	2015	Uttarakhand	1	1	1	1	1	25.97
Trochalopteron erythrocephalus	Leiothrichidae	2015	Uttarakhand	1	1	1	0	3	21.96
Trochalopteron erythrocephalus	Leiothrichidae	2015	Uttarakhand	1	1	1	1	1	33.61
Trochalopteron erythrocephalus	Leiothrichidae	2015	Uttarakhand	1	1	1	0	1	30.74
Trochalopteron erythrocephalus	Leiothrichidae	2014	Uttarakhand	1	1	1	0	3	22.16
Trochalopteron erythrocephalus	Leiothrichidae	2014	Uttarakhand	1	1	1	1	3	22.23
Trochalopteron erythrocephalus	Leiothrichidae	2015	Uttarakhand	1	1	1	1	3	23.86
Trochalopteron erythrocephalus	Leiothrichidae	2015	Uttarakhand	1	1	1	0	3	21.55
Trochalopteron lineatum	Leiothrichidae	2015	Uttarakhand	1	1	1	1	1	25.63
Trochalopteron lineatum	Leiothrichidae	2015	Uttarakhand	1	1	1	0	3	26.10
Trochalopteron variegatum	Leiothrichidae	2015	Uttarakhand	1	1	1	0	1	33.04
Trochalopteron variegatum	Leiothrichidae	2015	Uttarakhand	1	1	1	0	1	32.97
Trochalopteron variegatum	Leiothrichidae	2014	Uttarakhand	1	1	1	0	1	26.74
Turdoides affinis	Leiothrichidae	2015	Karnataka	1	1	1	1	3	22.40
Turdoides affinis	Leiothrichidae	2015	Karnataka	1	1	1	0	1	24.68

Turdoides striata	Leiothrichidae	2015	Uttarakhand	1	1	1	1	2	25.79
Turdoides striata	Leiothrichidae	2015	Uttarakhand	1	1	1	1	1	26.53
Turdoides striata	Leiothrichidae	2015	Uttarakhand	1	1	1	1	1	25.49
Turdoides striata	Leiothrichidae	2015	Uttarakhand	1	1	1	1	1	28.11
Turdus boulboul	Turdidae	2015	Uttarakhand	1	1	1	0	3	18.61
Turdus boulboul	Turdidae	2015	Uttarakhand	1	1	1	1	1	24.79
Turdus unicolor	Turdidae	2015	Uttarakhand	1	1	1	1	1	28.99
Yuhina flavicolli	Zosteropidae	2014	Uttarakhand	1	1	1	1	3	27.46
Zosterops palpebrosus	Zosteropidae	2014	Uttarakhand	1	1	1	1	2	28.65
Zosterops palpebrosus	Zosteropidae	2015	Uttarakhand	1	1	1	1	3	24.70
Zosterops palpebrosus	Zosteropidae	2014	Uttarakhand	1	1	1	1	1	26.22

SWEDEN

Species	Family	Year	Site	mic	qPCR	enz	nested	intensity	CT value
Acrocephalus palustris	Acrocephaliidae	2014	Sweden	0	0	0	0	0	-
Acrocephalus palustris	Acrocephaliidae	2014	Sweden	0	0	0	0	0	-
Acrocephalus palustris	Acrocephaliidae	2014	Sweden	0	0	0	0	0	-
Acrocephalus palustris	Acrocephaliidae	2014	Sweden	0	0	0	0	0	-
Acrocephalus palustris	Acrocephaliidae	2014	Sweden	0	0	0	0	0	-
Acrocephalus schoenobaenus	Acrocephaliidae	2014	Sweden	0	1	1	1	0	30.875
Acrocephalus schoenobaenus	Acrocephaliidae	2014	Sweden	0	1	1	0	0	34.84
Acrocephalus schoenobaenus	Acrocephaliidae	2014	Sweden	0	0	0	0	0	-
Acrocephalus schoenobaenus	Acrocephaliidae	2014	Sweden	0	0	0	0	0	-
Acrocephalus schoenobaenus	Acrocephaliidae	2014	Sweden	0	0	0	0	0	-
Acrocephalus schoenobaenus	Acrocephaliidae	2014	Sweden	0	0	0	0	0	-
Acrocephalus schoenobaenus	Acrocephaliidae	2014	Sweden	0	0	0	0	0	-
Acrocephalus schoenobaenus	Acrocephaliidae	2014	Sweden	0	0	0	0	0	-
Acrocephalus schoenobaenus	Acrocephaliidae	2014	Sweden	0	0	0	0	0	-
Acrocephalus schoenobaenus	Acrocephaliidae	2014	Sweden	0	0	0	0	0	-
Acrocephalus schoenobaenus	Acrocephaliidae	2014	Sweden	0	0	0	0	0	-
Acrocephalus schoenobaenus	Acrocephaliidae	2014	Sweden	0	0	0	0	0	-
Acrocephalus schoenobaenus	Acrocephaliidae	2014	Sweden	0	0	0	0	0	-
Acrocephalus schoenobaenus	Acrocephaliidae	2014	Sweden	0	0	0	0	0	-
Acrocephalus scirpaceus	Acrocephaliidae	2014	Sweden	0	1	0	1	0	33.43
Acrocephalus scirpaceus	Acrocephaliidae	2014	Sweden	0	1	0	0	0	34.485
Acrocephalus scirpaceus	Acrocephaliidae	2014	Sweden	0	0	0	0	0	-
Acrocephalus scirpaceus	Acrocephaliidae	2014	Sweden	0	0	0	0	0	-
Acrocephalus scirpaceus	Acrocephaliidae	2014	Sweden	0	0	0	0	0	-

Acrocephalus scirpaceus	Acrocephaliidae	2014	Sweden	0	0	0	0	0	-
Acrocephalus scirpaceus	Acrocephaliidae	2014	Sweden	0	0	0	0	0	-
Acrocephalus scirpaceus	Acrocephaliidae	2014	Sweden	0	0	0	0	0	-
Acrocephalus scirpaceus	Acrocephaliidae	2014	Sweden	0	0	0	0	0	-
Acrocephalus scirpaceus	Acrocephaliidae	2014	Sweden	0	0	0	0	0	-
Acrocephalus scirpaceus	Acrocephaliidae	2014	Sweden	0	0	0	0	0	-
Acrocephalus scirpaceus	Acrocephaliidae	2014	Sweden	0	0	0	0	0	-
Acrocephalus scirpaceus	Acrocephaliidae	2014	Sweden	0	0	0	0	0	-
Carduelis flammea	Fringillidae	2014	Sweden	0	0	0	0	0	-
Certhia familiaris	Certhiidae	2014	Sweden	0	0	0	0	0	-
Cyanistes caeruleus	Paridae	2014	Sweden	1	1	1	1	3	20.47
Cyanistes caeruleus	Paridae	2014	Sweden	1	1	1	1	2	20.98
Cyanistes caeruleus	Paridae	2014	Sweden	1	1	1	1	3	20.985
Cyanistes caeruleus	Paridae	2014	Sweden	1	1	1	1	2	21.645
Cyanistes caeruleus	Paridae	2014	Sweden	1	1	1	1	2	22.04
Cyanistes caeruleus	Paridae	2014	Sweden	1	1	1	1	1	22.185
Cyanistes caeruleus	Paridae	2014	Sweden	1	1	1	1	2	22.655
Cyanistes caeruleus	Paridae	2014	Sweden	1	1	1	1	2	22.775
Cyanistes caeruleus	Paridae	2014	Sweden	1	1	1	1	2	22.885
Cyanistes caeruleus	Paridae	2014	Sweden	1	1	1	1	2	22.96
Cyanistes caeruleus	Paridae	2014	Sweden	1	1	1	1	2	23.315
Cyanistes caeruleus	Paridae	2014	Sweden	1	1	1	1	1	23.51
Cyanistes caeruleus	Paridae	2014	Sweden	1	1	1	1	2	23.695
Cyanistes caeruleus	Paridae	2014	Sweden	1	1	1	1	2	23.88
Cyanistes caeruleus	Paridae	2014	Sweden	1	1	1	1	2	23.925
Cyanistes caeruleus	Paridae	2014	Sweden	1	1	1	1	2	24.115
Cyanistes caeruleus	Paridae	2014	Sweden	1	1	1	1	1	24.165
Cyanistes caeruleus	Paridae	2014	Sweden	1	1	1	1	2	24.195
Cyanistes caeruleus	Paridae	2014	Sweden	1	1	1	1	1	24.345
Cyanistes caeruleus	Paridae	2014	Sweden	1	1	1	1	1	24.345

Cyanistes caeruleus	Paridae	2014	Sweden	1	1	1	1	2	24.7
Cyanistes caeruleus	Paridae	2014	Sweden	1	1	1	1	1	25.24
Cyanistes caeruleus	Paridae	2014	Sweden	1	1	1	1	2	25.41
Cyanistes caeruleus	Paridae	2014	Sweden	0	1	1	1	0	27.395
Cyanistes caeruleus	Paridae	2014	Sweden	1	1	1	1	1	28.43
Cyanistes caeruleus	Paridae	2014	Sweden	0	1	1	1	0	29.92
Cyanistes caeruleus	Paridae	2014	Sweden	0	1	1	1	0	31.05
Cyanistes caeruleus	Paridae	2014	Sweden	0	1	1	1	0	33.425
Cyanistes caeruleus	Paridae	2014	Sweden	0	1	1	1	0	34.12
Cyanistes caeruleus	Paridae	2014	Sweden	0	0	0	0	0	-
Cyanistes caeruleus	Paridae	2014	Sweden	0	0	1	1	0	-
Emberiza citrinella	Emberizidae	2014	Sweden	1	1	1	1	3	17.995
Emberiza citrinella	Emberizidae	2014	Sweden	1	1	1	1	3	20.4
Emberiza schoeniclus	Emberizidae	2014	Sweden	0	0	0	0	0	-
Emberiza schoeniclus	Emberizidae	2014	Sweden	0	0	0	0	0	-
Emberiza schoeniclus	Emberizidae	2014	Sweden	0	0	0	0	0	-
Emberiza schoeniclus	Emberizidae	2014	Sweden	0	0	0	0	0	-
Emberiza schoeniclus	Emberizidae	2014	Sweden	0	0	0	0	0	-
Erithacus rubecula	Muscicapidae	2014	Sweden	1	1	1	1	1	25.555
Erithacus rubecula	Muscicapidae	2014	Sweden	0	1	0	1	0	30.9
Erithacus rubecula	Muscicapidae	2014	Sweden	0	1	1	1	0	32.63
Erithacus rubecula	Muscicapidae	2014	Sweden	0	1	1	1	0	33.98
Erithacus rubecula	Muscicapidae	2014	Sweden	0	0	0	0	0	-
Erithacus rubecula	Muscicapidae	2014	Sweden	0	0	0	0	0	-
Erithacus rubecula	Muscicapidae	2014	Sweden	0	0	0	1	0	-
Erithacus rubecula	Muscicapidae	2014	Sweden	0	0	0	0	0	-
Erithacus rubecula	Muscicapidae	2014	Sweden	0	0	0	0	0	-
Erithacus rubecula	Muscicapidae	2014	Sweden	0	0	0	0	0	-
Erithacus rubecula	Muscicapidae	2014	Sweden	0	0	0	0	0	-
Fringilla coelebs	Fringillidae	2014	Sweden	1	1	1	1	1	25.835

Hippolais icterina	Acrocephaliidae	2014	Sweden	1	1	1	1	2	22.74
Hippolais icterina	Acrocephaliidae	2014	Sweden	1	1	1	1	2	22.95
Locustella fluviatilis	Locustellidae	2014	Sweden	0	0	0	0	0	-
Locustella naevia	Locustellidae	2014	Sweden	0	0	0	1	0	-
Luscinia luscinia	Muscicapidae	2014	Sweden	0	1	1	1	0	29.2
Panurus biarmicus	Panuridae	2014	Sweden	0	0	0	0	0	-
Parus major	Paridae	2014	Sweden	1	1	1	1	3	20.63
Parus major	Paridae	2014	Sweden	1	1	1	1	3	20.63
Parus major	Paridae	2014	Sweden	1	1	1	1	2	22.195
Parus major	Paridae	2014	Sweden	1	1	1	1	1	24.385
Parus major	Paridae	2014	Sweden	1	1	1	1	1	25.03
Parus major	Paridae	2014	Sweden	1	1	1	1	1	26.05
Parus major	Paridae	2014	Sweden	0	1	1	1	0	26.655
Parus major	Paridae	2014	Sweden	1	1	1	1	1	26.665
Parus major	Paridae	2014	Sweden	1	1	1	1	1	27.19
Parus major	Paridae	2014	Sweden	0	1	1	1	0	28.935
Parus major	Paridae	2014	Sweden	0	1	1	1	0	30.435
Parus major	Paridae	2014	Sweden	0	1	1	1	0	30.91
Phoenicurus phoenicurus	Muscicapidae	2014	Sweden	0	0	0	0	0	-
Phylloscopus collybita	Phylloscopidae	2014	Sweden	1	1	1	1	1	24.155
Phylloscopus collybita	Phylloscopidae	2014	Sweden	1	1	1	1	1	25.46
Phylloscopus collybita	Phylloscopidae	2014	Sweden	0	1	1	1	0	27.405
Phylloscopus collybita	Phylloscopidae	2014	Sweden	0	1	1	1	0	34.74
Phylloscopus collybita	Phylloscopidae	2014	Sweden	0	0	0	0	0	-
Phylloscopus collybita	Phylloscopidae	2014	Sweden	0	0	0	0	0	-
Phylloscopus collybita	Phylloscopidae	2014	Sweden	0	0	0	0	0	-
Phylloscopus collybita	Phylloscopidae	2014	Sweden	0	0	0	0	0	-
Phylloscopus collybita	Phylloscopidae	2014	Sweden	0	0	0	0	0	-
Phylloscopus trochilus	Phylloscopidae	2014	Sweden	1	1	1	1	2	23.16
Phylloscopus trochilus	Phylloscopidae	2014	Sweden	0	1	1	1	0	26.2

Phylloscopus trochilus	Phylloscopidae	2014	Sweden	0	0	0	0	0	-
Phylloscopus trochilus	Phylloscopidae	2014	Sweden	0	0	0	0	0	-
Phylloscopus trochilus	Phylloscopidae	2014	Sweden	1	0	1	1	2	-
Poecile palustris	Paridae	2014	Sweden	1	1	1	1	1	25.055
Poecile palustris	Paridae	2014	Sweden	0	1	0	0	0	32.835
Poecile palustris	Paridae	2014	Sweden	1	1	1	1	1	34.955
Poecile palustris	Paridae	2014	Sweden	0	0	0	0	0	-
Poecile palustris	Paridae	2014	Sweden	0	0	0	0	0	-
Prunella modularis	Prunellidae	2014	Sweden	0	1	0	0	0	33.5
Prunella modularis	Prunellidae	2014	Sweden	0	1	0	1	0	34.75
Prunella modularis	Prunellidae	2014	Sweden	0	0	0	0	0	-
Prunella modularis	Prunellidae	2014	Sweden	0	0	0	0	0	-
Prunella modularis	Prunellidae	2014	Sweden	0	0	0	0	0	-
Prunella modularis	Prunellidae	2014	Sweden	0	0	0	0	0	-
Prunella modularis	Prunellidae	2014	Sweden	0	0	0	0	0	-
Prunella modularis	Prunellidae	2014	Sweden	0	0	0	0	0	-
Prunella modularis	Prunellidae	2014	Sweden	0	0	0	0	0	-
Prunella modularis	Prunellidae	2014	Sweden	0	0	1	1	0	-
Prunella modularis	Prunellidae	2014	Sweden	0	0	0	0	0	-
Prunella modularis	Prunellidae	2014	Sweden	0	0	0	0	0	-
Prunella modularis	Prunellidae	2014	Sweden	0	0	0	0	0	-
Prunella modularis	Prunellidae	2014	Sweden	0	0	0	0	0	-
Prunella modularis	Prunellidae	2014	Sweden	0	0	0	0	0	-
Saxicola rubetra	Muscicapidae	2014	Sweden	1	1	1	1	2	21.195
Saxicola rubetra	Muscicapidae	2014	Sweden	0	1	1	1	0	27.77
Saxicola rubetra	Muscicapidae	2014	Sweden	1	1	1	1	1	28.94
Saxicola rubetra	Muscicapidae	2014	Sweden	0	1	0	1	0	29.95
Saxicola rubetra	Muscicapidae	2014	Sweden	0	1	0	0	0	38.105
Saxicola rubetra	Muscicapidae	2014	Sweden	0	0	0	0	0	-
Saxicola rubetra	Muscicapidae	2014	Sweden	0	0	0	1	0	-

Saxicola rubetra	Muscicapidae	2014	Sweden	0	0	0	0	0	-
Saxicola rubetra	Muscicapidae	2014	Sweden	0	0	0	0	0	-
Saxicola rubetra	Muscicapidae	2014	Sweden	0	0	0	0	0	-
Sitta europaea	Sittidae	2014	Sweden	1	1	1	1	2	24.755
Sitta europaea	Sittidae	2014	Sweden	0	0	0	0	0	-
Sylvia atricapilla	Sylviidae	2014	Sweden	1	1	1	1	3	21.27
Sylvia atricapilla	Sylviidae	2014	Sweden	1	1	1	1	1	22.33
Sylvia atricapilla	Sylviidae	2014	Sweden	1	1	1	1	2	23.105
Sylvia atricapilla	Sylviidae	2014	Sweden	1	1	1	1	2	23.11
Sylvia atricapilla	Sylviidae	2014	Sweden	1	1	0	0	2	23.18
Sylvia atricapilla	Sylviidae	2014	Sweden	1	1	1	1	2	23.18
Sylvia atricapilla	Sylviidae	2014	Sweden	1	1	1	1	2	23.4
Sylvia atricapilla	Sylviidae	2014	Sweden	1	1	1	1	2	23.56
Sylvia atricapilla	Sylviidae	2014	Sweden	1	1	1	1	1	25.31
Sylvia atricapilla	Sylviidae	2014	Sweden	1	1	1	1	2	26.525
Sylvia borin	Sylviidae	2014	Sweden	1	1	1	1	2	21.2
Sylvia borin	Sylviidae	2014	Sweden	1	1	1	1	2	21.425
Sylvia borin	Sylviidae	2014	Sweden	1	1	1	1	1	24.415
Sylvia borin	Sylviidae	2014	Sweden	1	1	1	1	1	24.735
Sylvia borin	Sylviidae	2014	Sweden	0	1	1	1	0	25.1
Sylvia borin	Sylviidae	2014	Sweden	1	1	1	1	1	27.565
Sylvia borin	Sylviidae	2014	Sweden	0	1	1	1	0	29.93
Sylvia borin	Sylviidae	2014	Sweden	0	0	0	0	0	-
Sylvia communis	Sylviidae	2014	Sweden	1	1	1	1	3	18.61
Sylvia communis	Sylviidae	2014	Sweden	1	1	1	1	2	24.305
Sylvia communis	Sylviidae	2014	Sweden	1	1	1	1	1	25.19
Sylvia communis	Sylviidae	2014	Sweden	0	1	1	1	0	26.35
Sylvia communis	Sylviidae	2014	Sweden	1	1	1	1	1	26.77
Sylvia communis	Sylviidae	2014	Sweden	0	1	1	1	0	27.185
Sylvia communis	Sylviidae	2014	Sweden	1	1	1	1	1	27.46

Sylvia communis	Sylviidae	2014	Sweden	1	1	1	1	1	27.66
Sylvia communis	Sylviidae	2014	Sweden	0	1	1	0	0	29.465
Sylvia communis	Sylviidae	2014	Sweden	0	1	0	1	0	32.99
Sylvia communis	Sylviidae	2014	Sweden	0	1	0	1	0	33.36
Sylvia communis	Sylviidae	2014	Sweden	0	0	0	0	0	-
Sylvia communis	Sylviidae	2014	Sweden	0	0	0	0	0	-
Sylvia communis	Sylviidae	2014	Sweden	0	0	0	0	0	-
Sylvia communis	Sylviidae	2014	Sweden	0	0	0	0	0	-
Sylvia communis	Sylviidae	2014	Sweden	0	0	0	0	0	-
Sylvia communis	Sylviidae	2014	Sweden	0	0	0	0	0	-
Sylvia communis	Sylviidae	2014	Sweden	0	0	0	0	0	-
Sylvia curruca	Sylviidae	2014	Sweden	0	0	0	0	0	-
Troglodytes troglodytes	Troglodytidae	2014	Sweden	1	1	1	1	1	21.535
Troglodytes troglodytes	Troglodytidae	2014	Sweden	0	0	0	0	0	-
Troglodytes troglodytes	Troglodytidae	2014	Sweden	0	0	0	1	0	-
Turdus philomelus	Turdidae	2014	Sweden	1	1	1	1	2	24.005
Turdus philomelus	Turdidae	2014	Sweden	1	1	1	1	2	25.165

Appendix XX – All samples with blood smears were examined with four different screening protocols, and the result for the screening of parasites is represented as presence (1) and absence (0) in the table here. Presence or absence of multiple infections has also been mentioned. The intensity of the parasites as observed through microscopy and the corresponding CT value for each of the samples has been listed.

.* Abbreviations in Appendix are as follows: mic – Microscopy, enz – Restriction- enzyme based assay, nested – Nested protocol, and qPCR– quantitative PCR.
